# Female Patients with Myalgic Encephalomyelitis/Chronic Fatigue Syndrome or Idiopathic Chronic Fatigue: Comparison of Responses to a Two-Day Cardiopulmonary Exercise Testing Protocol

**DOI:** 10.3390/healthcare9060682

**Published:** 2021-06-05

**Authors:** C. (Linda) M. C. van Campen, Frans C. Visser

**Affiliations:** Stichting Cardiozorg, Planetenweg 5, 2132 HN Hoofddorp, The Netherlands; fransvisser@stichtingcardiozorg.nl

**Keywords:** chronic fatigue syndrome, cardiopulmonary exercise testing, VO_2_ peak, ventilatory threshold, VO_2_AT, RER, myalgic encephalitis, workload, idiopathic chronic fatigue

## Abstract

Introduction: Multiple studies have shown that peak oxygen consumption is reduced in the majority of ME/CFS patients, using the golden standard for measuring exercise intolerance: cardiopulmonary exercise testing (CPET). A 2-day CPET protocol has shown different results on day 2 in ME/CFS patients compared to sedentary controls. No comparison is known between ME/CFS and idiopathic chronic fatigue (ICF) for 2-day CPET protocols. We compared ME/CFS patients with patients with chronic fatigue who did not fulfil the ME/CFS criteria in a male population and hypothesized a different pattern of response would be present during the 2nd day CPET. Methods: Fifty-one female patients with ICF completed a 2-day CPET protocol and were compared to an age/sex-matched group of 50 female ME/CFS patients. Measures of oxygen consumption (VO_2_), heart rate (HR), systolic and diastolic blood pressure, workload (Work), and respiratory exchange ratio (RER) were collected at maximal (peak) and ventilatory threshold (VT) intensities. Results: Baseline characteristics for both groups were similar for age, BMI, BSA, and disease duration. A significance difference was present in the number of patients with fibromyalgia (seven ME/CFS patients vs zero ICF patients). Heart rate at rest and the RER did not differ significantly between CPET 1 and CPET 2. All other CPET parameters at the ventilatory threshold and maximum exercise differed significantly (p-value between 0.002 and <0.0001). ME/CFS patients showed a deterioration of performance on CPET2 as reflected by VO_2_ and workload at peak exercise and ventilatory threshold, whereas ICF patients showed improved performance on CPET2 with no significant change in peak workload. Conclusion: This study confirms that female ME/CFS patients have a reduction in exercise capacity in response to a second day CPET. These results are similar to published results in female ME/CFS populations. Patients diagnosed with ICF show a different response on day 2, more similar to sedentary and healthy controls.

## 1. Introduction

Myalgic encephalomyelitis/chronic fatigue syndrome (ME/CFS) is a serious and potentially disabling chronic disease [1,2,3,4]. An important symptom of patients with ME/CFS is exercise intolerance along with prolonged recovery from exercise (physical, mental, emotional, as well as also orthostatic stress) and post-exertional exacerbation of ME/CFS symptoms [4], termed post-exertional malaise (PEM) [5,6]. The pathophysiology of the exercise intolerance is not exactly known but involves both metabolic abnormalities of skeletal muscles as well as central nervous system abnormalities [5,7,8,9,10,11,12].

The golden standard for measuring the severity of physical activity intolerance is considered to be cardiopulmonary exercise testing (CPET). Multiple studies have shown that peak oxygen consumption is reduced in the majority of ME/CFS patients [13,14,15,16,17,18,19,20,21,22,23,24]. However, studies have also shown that a single CPET may show in ME/CFS patients that peak VO_2_ values can be similar to or only slightly lower than those of healthy sedentary controls [13,14,15,16,17,18,19,20,21,22,23,24]. Several studies have confirmed that after a 2-day CPET protocol, with two CPET separated by 24 h, ME/CFS patients have significantly lower VO_2_ and workload parameters on day 2 (CPET 2) than on day 1 (CPET 1) [17,20,21,22,25]. In contrast, sedentary controls have unaltered or slightly improved VO_2_ and workload [20,21,22,25,26]. No 2-day CPET protocol was performed with patients with idiopathic chronic fatigue.

Although the single-day CPET study by Vermeulen et al. [23] included participants with idiopathic chronic fatigue along with ME/CFS patients and healthy controls, no 2-day CPET protocol has examined patients with idiopathic chronic fatigue. We hypothesized that patients diagnosed with idiopathic chronic fatigue, who do not fulfill ME/CFS criteria, might have a different pathophysiology and response to a 2-day CPET protocol. Because of the lack of information on exercise intolerance/performance in patients with idiopathic chronic fatigue in 2-day CPET protocols, the aim of this study was to examine the effect of a 2-day CPET protocol in female ME/CFS patients and in female ICF patients.

## 2. Materials and Methods

### 2.1. Participants

To test our hypothesis we selected female patients fulfilling the criteria of ME/CFS, comparing them with female patients not fulfilling the criteria and therefore were diagnosed with idiopathic chronic fatigue (ICF) [1,3]. We elected to answer the hypothesis by studying only female patients. This was due to the fact that there are sex differences in peak oxygen consumption, and there may be possible sex differences in the clinical phenotype of the disease [27,28,29,30]. We reported results on males with ME/CFS and ICF separately. No important comorbidities or alternative explanations for the symptomatology were present. Female ME/CFS patients graded as having severe ME/CFS according to International Consensus Criteria (ICC) were excluded from this analysis, as none of the ICF patients of the control group had a disease of comparable severity. In order to report on the stated hypothesis and aim of the study, exercise intolerance information as derived from a 2-day CPET protocol was subtracted from the clinical database on female patients analyzed for fatigue and complaints of exercise intolerance (see patient flow figure in Appendix A)

All patients underwent a detailed clinical history to establish the diagnosis of ME/CFS according to the ME criteria [1] and CFS criteria of Fukuda [3]. Of the initially 235 female patients undergoing CPET for clinical reasons for determining exercise intolerance, between June 2010 and October 2019, 65 patients did not fulfill the ME/CFS criteria and were therefore diagnosed with idiopathic chronic fatigue. Fifty-seven ME/CFS female patients only had a single CPET and 38 patients had more than one test, but not on 2 consecutive days. Female ME/CFS patients clinically graded as having severe ME/CFS according to ICC were excluded from this analysis [1]. This was done in order to have the patient and control group of similar clinical severity, as none of the ICF patients of the control group had a clinically severe disease. Twenty-four clinically severe female ME/CFS patients were excluded, leaving 51 female patients with data from a 2-day CPET protocol available for analysis. In this period, 50 female patients with idiopathic chronic fatigue underwent a 2-day CPET protocol to quantify exercise intolerance. They were considered the control group for this analysis.

All patients gave written informed consent to analyze their data. The use of clinical data for descriptive studies was approved by the ethics committee of the Slotervaart Hospital, the Netherlands

### 2.2. Cardiopulmonary Exercise Testing (CPET)

Patients underwent a symptom-limited exercise test on a cycle ergometer (Excalibur, Lode, Groningen, the Netherlands) according to a previously described protocol [31,32,33]. A ramp workload protocol was used, varying between 10 and 30 watt/min increases, depending on sex, age, and expected exercise intolerance. Volume of oxygen consumption (VO_2_), volume of carbon dioxide release (VCO_2_), and oxygen saturation were continuously measured (Cortex, Procare, the Netherlands) and displayed on screen using Metasoft software (Cortex, Biophysic Gmbh, Germany). An ECG was continuously recorded and blood pressure was measured continuously using the Nexfin device (BMEYE, Amsterdam, the Netherlands) [34]. Cycle seat height was positioned to approximately 175° of knee extension, and the same seat height was used for both tests. Expired gases were collected breath-by-breath through a two-way breathing valve and analyzed using open circuit spirometry. The metabolic measurement system (Cortex, Biophysic Gmbh, Germany) was gas, pressure, and volume calibrated before each test with ambient air, standard gases of known concentrations, and a 3 L calibration syringe [35,36]. The ventilatory threshold (VT), a measure of the anaerobic threshold, was identified from expired gases using the V-Slope algorithm [37]. The ventilatory or anaerobic threshold is the exercise intensity at which metabolism, due to a lack of a sufficient amount of oxygen for energy production, transitions towards increased anaerobic energy production. The same experienced cardiologist supervised the test and performed visual assessment and confirmation of the algorithm-derived VT. Testing took place in a controlled environment with a temperature range of 20–24 °C and 15–60% relative humidity. Patients were encouraged by standard phrases each minute to perform as maximally as possible. The mean of the VO_2_ measurements of the last 15 s before ending the exercise (peak VO_2_) was taken. VO_2_ at the peak and at the VT as well as the heart rate (HT) at peak exercise were expressed as a percentage of the normal values of a population study: %peak VO_2_, %VT VO_2_ and %peak HR [38]. Moreover, the mean respiratory exchange ratio (RER; VCO_2_/VO_2_) of the last 15 s was calculated. Immediately after the test, the attending cardiologist noted the primary reason for the termination of the exercise test.

According to a study of Parsaik et al. from 2012 on exercise intolerance/deconditioning results from the gold standard CPET, the percent predicted VO_2_ peak indicates fitness or the amount of exercise tolerance. The authors defined no deconditioning if the percent predicted VO_2_ was ≥85% and defined moderate deconditioning if the percent predicted VO_2_ was <85% [39].

### 2.3. Statistical Analysis

Data were analyzed using the statistical package of Graphpad Prism version 8.4.2 (Graphpad software, La Jolla, CA, USA). All continuous data were tested to identify outliers with the Grubb’s test and tested for normal distribution using the D’Agostino-Pearson omnibus normality test, and were presented as mean with the standard deviation (SD) or as median with the inter quartile range (IQR), where appropriate. Nominal data (fibromyalgia and severity/disability) were compared using the chi-square test. For fibromyalgia, this was achieved in a yes present or no absent comparison. Disease severity included mild or moderate and was graded clinically. For the chi-square in a 2 × 2 table, it was tested for ME/CFS versus ICF and numbers of mild vs. moderate disease. For continuous data, groups were compared using the paired t-test/Wilcoxon matched pairs signed rank test when comparing within group as for comparing day 1 and 2. For continuous data, groups were compared using the unpaired t-test/Mann–Whitney test when comparing two separate groups for f.i. day 1 variables or day 2 variables. A *p*-value of <0.01 was considered significantly different.

## 3. Results

**Figure 1:**
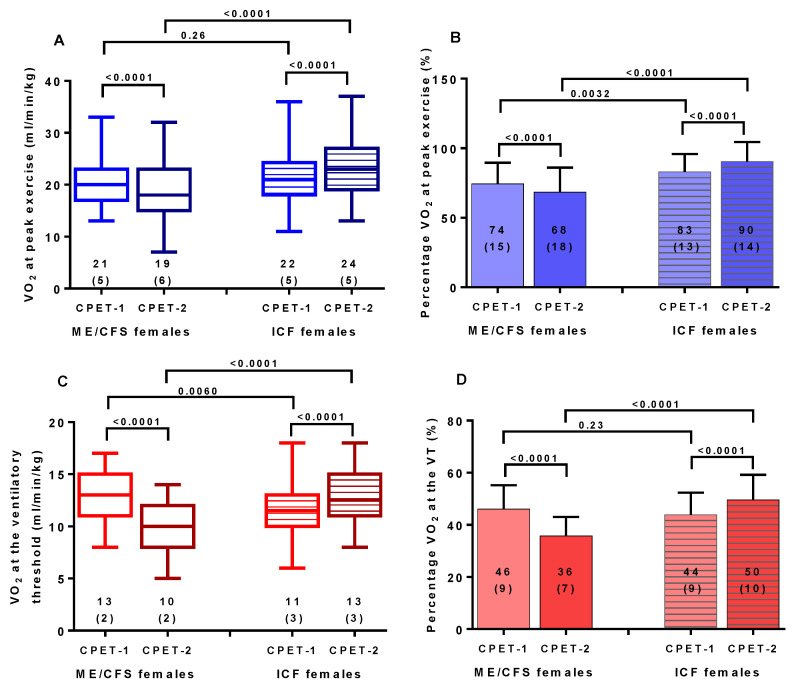
Peak exercise values and values at the ventilatory threshold for CPET-1 and CPET-2 (panel (**A**): peak oxygen consumption, panel (**B**): percent predicted peak oxygen consumption, panel (**C**): oxygen consumption at the ventilatory threshold, and panel (**D**): percent predicted oxygen consumption at the ventilatory threshold) for female ME/CFS and female ICF patients. CPET: cardiopulmonary exercise test; VT: ventilatory threshold; VO_2_: volume of oxygen consumption; ME/CFS: myalgic encephalomyelitis/chronic fatigue syndrome; ICF: idiopathic chronic fatigue.

**Figure 2:**
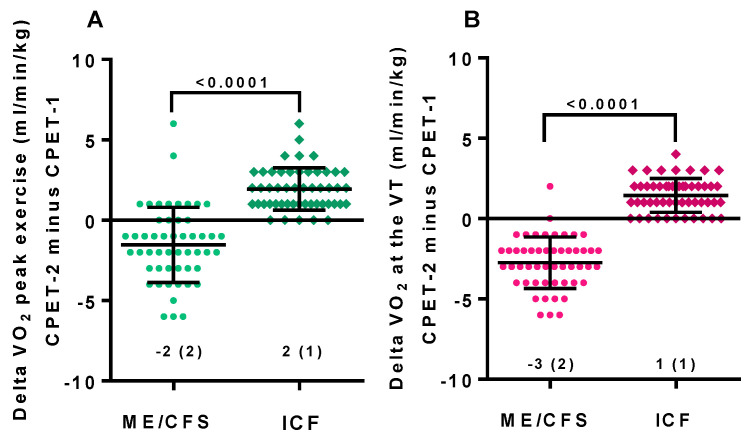
The range of absolute differences for peak VO_2_ (**A**) and VO_2_ at the ventilatory threshold (**B**) for female ME/CFS and female ICF patients. CPET: cardiopulmonary exercise test; ME/CFS: myalgic encephalomyelitis/chronic fatigue syndrome; ICF: idiopathic chronic fatigue; VO_2_: volume of oxygen consumption; VT: ventilatory threshold.

Table 1 shows the baseline characteristics of the study participants. There was a higher proportion of fibromyalgia in the patient group with ME/CFS: 25/51 (49%) compared to 7/50 (14%) for the idiopathic chronic fatigue patient group. This was statistically significantly different (*p* < 0.001). Age, height, weight, body surface area, body mass index, disease severity grade, and disease duration were comparable in both groups. Table 2 shows the parameters of the CPET of day 1 and day 2 compared in ME/CFS females and in ICF females. For both groups, heart rate at rest was not significantly different. For female ME/CFS patients, all CPET parameters declined significantly comparing day 1 and day 2 (*p*-value all <0.0001). For the female ICF patients also, no significant difference was found for peak workload between day 1 and day 2; other exercise parameters increased significantly from day 1 to day 2. Figure 1 shows the peak VO_2_ values and VO_2_ at the ventilatory threshold for ME/CFS and ICF females for the 2-day CPET protocol. All were highly significantly different (*p* < 0.0001). Where CPET parameters decreased on day 2 in ME/CFS patients, the same values increased on day 2 in ICF patients: a completely different pattern. Figure 2 shows the range of differences in VO_2_ at peak exercise (CPET-2 minus CPET-1) and VO_2_ at the ventilatory threshold. For both parameters a highly significant difference was shown (both *p* < 0.0001), but a certain spread of values was also present for both studied patient groups.

**Figure 3:**
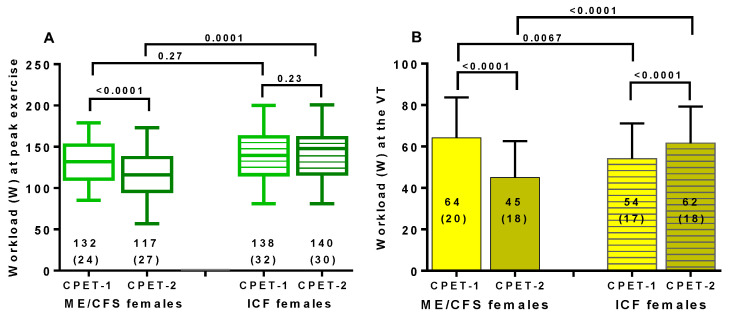
The workload at peak exercise for CPET-1 and CPET-2 (panel **A**) and at the ventilatory threshold for CPET-1 and CPET-2 (panel **B**). On the left side, the values of day 1 and day 2 are shown for female ME/CFS patients (clear boxes/columns); on the right side, the values of day 1 and day 2 are shown for female ICF patients (striped boxes/columns). Workload at peak exercise for CPET-1 and CPET-2 (panel **A**) and at the ventilatory threshold for CPET-1 and CPET-2 (panel **B**). Legend Figure 3: CPET: cardiopulmonary exercise test; ME/CFS: myalgic encephalomyelitis/chronic fatigue syndrome; ICF: idiopathic chronic fatigue; VT: ventilatory threshold.

**Figure 4:**
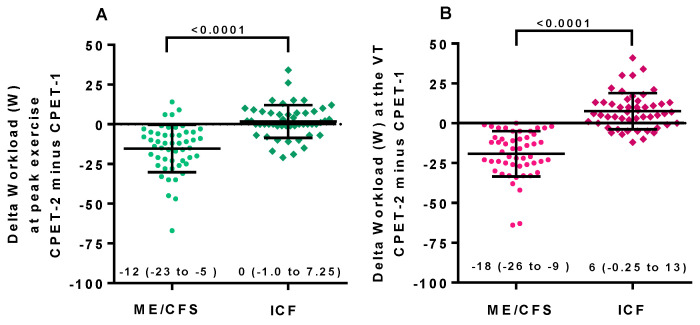
The range of absolute differences for peak workload (**A**) and workload at the ventilatory threshold (**B**) for female ME/CFS and female ICF patients. Legend Figure 4: CPET: cardiopulmonary exercise test; ME/CFS: myalgic encephalomyelitis/chronic fatigue syndrome; ICF: idiopathic chronic fatigue; VT: ventilatory threshold.

Figure 3 shows the changes in workload at peak exercise and at the ventilatory threshold for both ME/CFS and ICF patients. Day 1 and day 2 differed significantly in both (*p* all < 0.0001). In ME/CFS patients, a higher workload at the ventilatory threshold on day 1 was found (*p* < 0.05), compared to ICF patients. This difference was larger comparing day 2 results between ME/CFS and ICF patients (*p* = 0.0005). Figure 4 shows the difference in both workload measures when day 1 and day 2 were compared in the two groups. Both differed significantly (*p*-value both < 0.0001).

Table 3 shows the parameters of the CPET of day 1 (left columns) compared between ME/CFS and ICF females and the parameters of the CPET of day 2 (right columns) compared between ME/CFS and ICF females. Heart rate, workload, and RER were not significantly different at day 1 between ME/CFS and ICF patients. With an RER of >1, a maximal effort was present in all studied patients. At the maximal exercise on day one, no significant difference for peak VO_2_ was found. For the % predicted VO_2_ between ME/CFS and ICF patients at day 1, a significant difference was measured. At the VO_2_ at the ventilatory threshold, heart rate and workload were higher in ME/CFS patients compared to ICF patients (*p*-value varying between 0.007 and 0.0003). On day 2 CPET, more differences were seen: at the ventilatory threshold and at peak exercise, with higher values of peak VO_2_, percent predicted peak, peak workload for ICF females compared to ME/CFS females (*p*-value ranging from 0.0006 to < 0.0001). Furthermore, more differences on day 2 at the ventilatory threshold were seen, with higher values of VO_2_ at the ventilatory threshold, percent predicted VO_2_-VT, and VT workload for ICF females compared to ME/CFS females (*p*-value all < 0.0001).

Table 4 shows the number of females with ME/CFS on the left and ICF on the right for no deconditioning and for moderate deconditioning as determined by CPET. The results are shown for both CPET-1 and CPET-2. The table shows that for ME/CFS patients, the minority (22%) are not deconditioned as defined in past literature, whereas for ICF patients it is about 50/50 (48%). What the table further clarifies is that for ME/CFS, more patients are of moderate deconditioning on day 2 compared to day 1. For ICF patients, this is completely opposite: more patients have no deconditioning compared to moderate deconditioning.

## 4. Discussion

The main finding of this study is that a distinctive difference in response on especially CPET day 2 is present when comparing the ME/CFS females with the ICF females. Where peak exercise values as well as values at the ventilatory threshold in ME/CFS deteriorate on day 2, the response in ICF patients with respect to peak exercise values and values at the ventilatory threshold is more similar to the response of sedentary controls on the second day of a 2-day CPET protocol.

A two-day CPET protocol in ME/CFS patients shows a unique feature of the disease: reduced CPET parameters at the VO_2_ peak and at the ventilatory threshold at the second day of the protocol, which is in contrast to the VO_2_ data found in sedentary controls [20,21,22,25,26]. The present study is the first study to reveal that patients with the diagnosis of idiopathic chronic fatigue show a different response pattern on a 2-day CPET protocol than ME/CFS patients do, suggesting the abnormalities found in ME/CFS patients on day 2 and the difference with day 1 are indeed a unique feature of the disease. The findings of the lower VO_2_ at peak exercise on the second day in ME/CFS patients, compared to sedentary controls, makes it unlikely that this phenomenon is due to deconditioning [21,40], and suggests metabolic abnormalities. It may represent an early sign of post-exertional malaise (PEM) [4], a cardinal feature of the disease. The general improvement on day 2 for patients with idiopathic chronic fatigue, who are without the symptom of post-exertional malaise and who show a pattern similar to sedentary and healthy controls, makes the suggestion that the findings in ME/CFS are disease-specific and a sign of post-exertional malaise, a cardinal symptom of the disease. In literature, no 2-day CPET protocols have been reported on idiopathic chronic fatigue patients.

### 4.1. Cardiopulmonary Exercise Testing in Idiopathic Chronic Fatigue: Comparison to Literature

As far as we know, only one study—the study by Vermeulen et al. from 2014—reported on a large sample of patients (females and males with idiopathic chronic fatigue), but the study showed only one-day CPET test results. Three groups were described in this manuscript: ME/CFS patients, ICF patients, and healthy controls were included and analyzed [23]. Vermeulen et al. reported a single-day CPET protocol comparison of 11 female healthy controls, 178 female ME/CFS patients, and 172 female ICF patients. This paragraph describes the relevant for our study data from the study of Vermeulen et al. Both ME/CFS and ICF females had lower peak VO_2_ values than healthy female subjects. At rest, no differences were found in baseline criteria and CPET data. At the ventilatory threshold, an ordinary one-way ANOVA was significant for VO_2_: 10.9 (2.6) mL/min/kg for female ME/CFS patients, 11.6 (2.7) mL/min/kg for female ICF patients, and 13.7 (3.6) for healthy female subjects (ANOVA *p* = 0.0001). No difference was found for the HR at the VT: 112 (15.6), 112.3 (15.2), and 113.3 (8.7), respectively (ANOVA *p* = 0.955), or RER 0.84 (0.07), 0.85 (0.08), and 0.81 (0.06) (ANOVA *p* = 0.216). At peak exercise, an ordinary one-way ANOVA was significant for VO_2_: 20.3 (5.0) mL/min/kg for female ME/CFS patients, 22.2 (5.3) mL/min/kg for female ICF patients, and 27.4 (7.2) for healthy female subjects (*p* < 0.0001). This was similar for percent predicted VO_2_: 80.6 (17.9)%, 83.1 (18.0)%, and 105.4 (18.8%), respectively (ANOVA *p* < 0.0001). Peak HR was not significantly different: 158.4 (19.3) bpm, 159.6 (18.6) bpm, and 164.1 (11.3) bpm, respectively (ANOVA *p* = 0.250), as well as RER: 1.16 (0.10), 1.18 (0.10), and 1.20 (0.11), respectively (ANOVA *p* = 0.094). No post hoc statistical information was present considering the significant difference of ME/CFS and ICF results between those groups. In the present study, VO_2_ at the ventilatory threshold for day 1 was 13 mL/min/kg for ME/CFS females and 11 mL/min/kg for ICF females, similar to the results of the study of Vermeulen et al. [23]. The peak VO_2_ was between 21 and 22 mL/min/kg for ME/CFS and ICF females, which was in the same range in our study and as described in the study of Vermeulen et al.

### 4.2. Cardiopulmonary Exercise Testing 2-Day Protocols: Comparison to Literature

Comparisons of values from previously reported 2-day CPET protocols have been reported in two of our recent studies on male ME/CFS and female ME/CFS patients, respectively [31,32], so we will focus on a comparison of the patients with idiopathic chronic fatigue from the present study with the sedentary controls in the 2-day CPET protocol studies, as the pattern of improvement seen in the present study seems to resemble those of sedentary controls. To the best of our knowledge, no 2-day protocols with patients diagnosed as having ICF have been executed and reported.

### 4.3. Limitations

No female sedentary controls were included for comparison in this study. Information on sedentary/healthy controls is described in literature by other research groups. As a control group, we used females not fulfilling ME/CFS criteria, resulting in having a diagnosis of idiopathic chronic fatigue (ICF). Second, this was not a prospective trial, as most patients underwent consecutive-day CPET for clinical management reasons in determining exercise intolerance. Third, differences between the previously discussed studies and the present study might be in the demographic characteristics and illness severity of the study population, but also in the exact methodology of the CPET used in the different study centers. Finally, reference values for predicted VO_2_ can differ between studies as well.

## 5. Conclusions

This study in ME/CFS females compared with ICF females shows that exercise capacity expressed in peak VO_2_, VO_2_ at the ventilatory threshold, and workload both at peak and at the ventilatory threshold show a different pattern from day 1 to day 2. In ME/CFS females, values decreased significantly on day 2 compared to day 1. In ICF females, values increased significantly on day 2 compared to day 1, suggesting that despite fatigue complaints of similar severity, a different disease is present and ICF might be trainable, whereas ME/CFS is not.

## Figures and Tables

**Table 1:** Baseline characteristics.

	Female ME/CFS (n = 51)	Female ICF (n = 50)	*p*-Value
Age (years)	40 (9)	40 (10)	0.68
Height (cm)	169 (6)	171 (7)	0.20
Weight (kg)	68 (10)	73 (14)	0.04
BMI (kg/m^2^)	1.4 (0.2)	1.4 (0.2)	0.04
BSA (m^2^)	23.9 (3.3)	25.1 (4.2)	0.13
Disease duration (years)	13 (9)	11 (7)	0.13
Disease severity grade ½ *	29/22 (57/43%)	36/14 (72/28%)	0.11
Fibromyalgia present *	25	7	<0.0005

CFS: chronic fatigue syndrome; BMI: body mass index (DuBois formula); BSA: body surface area. ICF: idiopathic chronic fatigue; ME: myalgic encephalomyelitis; mean (SD), analysis with unpaired *t*-test; * analysis with chi-square.

**Table 2:** CPET-1 and CPET-2 variables for female ME/CFS patients (left side) and female ICF patients (right side).

	ME/CFS Females (n = 51)			ICF Females (n = 50)		
**Peak Exercise**	**CPET-1**	**CPET-2**	***p*-Value**	**CPET-1**	**CPET-2**	***p*-Value**
VO_2_ peak (ml/min/kg)	21 (5)	19 (6)	< 0.0001	22 (5)	24 (5)	< 0.0001
%pred VO_2_ peak	74 (15)	68 (18)	< 0.0001	83 (13)	90 (14)	< 0.0001
HR rest (bpm)	87 (11)	87 (11)	0.80	88 (13)	88 (13)	0.82
HR peak (bpm)	156 (17)	149 (20)	< 0.0001	156 (18)	162 (16)	< 0.0001
Workload peak (watts)	132 (24)	117 (27)	< 0.0001	138 (31)	140 (30)	0.23
RER peak	1.1 (0.1)	1.1 (0.1)	0.03	1.2 (0.1)	1.1 (0.1)	0.80
**Ventilatory Threshold**	**CPET-1**	**CPET-2**	***p*-Value**	**CPET-1**	**CPET-2**	***p*-Value**
VO_2_ VT (ml/min/kg)	13 (2)	10 (2)	< 0.0001	11 (2)	13 (3)	< 0.0001
%pred VO_2_ VT	46 (9)	36 (7)	< 0.0001	44 (8)	50 (10)	< 0.0001
HR VT (bpm)	117 (13)	106 (12)	< 0.0001	107 (14)	113 (15)	< 0.0001
Workload VT (watts)	64 (19)	45 (17)	< 0.0001	54 (17)	62 (17)	< 0.0001

CFS: chronic fatigue syndrome; ICF: idiopathic chronic fatigue; ME: myalgic encephalomyelitis; VT: ventilatory threshold; CPET: cardiopulmonary exercise test; HR: heart rate; pred: predicted; RER: respiratory exchange ratio; VO_2_: volume of oxygen consumption.

**Table 3:** CPET-1 variables in ME/CFS compared to ICF females (left side) and CPET-2 variables in ME/CFS compared to ICF females (right side).

	CPET Day 1			CPET Day 2		
**Peak Exercise**	**ME/CFS**	**ICF**	***p*-Value**	**ME/CFS**	**ICF**	***p*-Value**
VO_2_ peak (ml/min/kg)	21 (5)	22 (5)	0.26	19 (6)	24 (5)	< 0.0001
%pred VO_2_ peak	74 (15)	83 (13)	0.003	68 (18)	90 (14)	< 0.0001
HR rest (bpm)	87 (11)	88 (13)	0.62	87 (11)	88 (13)	0.60
HR peak (bpm)	156 (17)	156 (18)	0.89	149 (20)	162 (16)	0.0006
Workload peak (watts)	132 (24)	138 (31)	0.28	117 (27)	140 (30)	0.0001
RER peak	1.1 (0.1)	1.2 (0.1)	0.03	1.1 (0.1)	1.2 (0.1)	0.0005
**Ventilatory Threshold**	**ME/CFS**	**ICF**	***p*-Value**	**CPET-1**	**CPET-2**	***p*-Value**
VO_2_ VT (ml/min/kg)	13 (2)	11 (2)	0.006	10 (2)	13 (3)	< 0.0001
%pred VO_2_ VT	46 (9)	44 (8)	0.23	36 (7)	50 (10)	< 0.0001
HR VT (bpm)	117 (13)	107 (14)	0.0003	106 (12)	113 (15)	0.02

CFS: chronic fatigue syndrome; ICF: idiopathic chronic fatigue; ME: myalgic encephalomyelitis; VT: ventilatory threshold; CPET: cardiopulmonary exercise test; HR: heart rate; pred: predicted; RER: respiratory exchange ratio; VO_2_: volume of oxygen consumption.

**Table 4:** CPET-1 and CPET-2 variables for female ME/CFS patients (left side) and female ICF patients (right side) with respect to “fitness”/ deconditioning.

	ME/CFS Females (n = 51)		ICF Females (n = 50)	
Peak Exercise	CPET-1	CPET-2	CPET-1	CPET-2
%pred VO_2_ peak ≥ 85% No deconditioning n=	9 (22%)	6 (16%)	24 (48%)	31 (66%)
%pred VO_2_ peak < 85% Moderate deconditioning n=	40 (78%)	43 (84%)	26 (52%)	17 (34%)

CFS: chronic fatigue syndrome; ICF: idiopathic chronic fatigue; ME: myalgic encephalomyelitis; CPET: cardiopulmonary exercise test; HR: heart rate; pred: predicted; VO_2_: volume of oxygen consumption.

**Figure A1:**
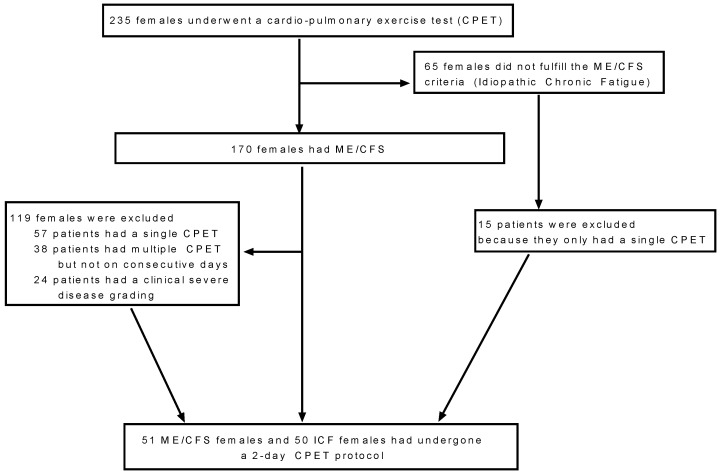
Patient flow. CPET: cardiopulmonary exercise test; ME/CFS: myalgic encephalomyelitis/chronic fatigue syndrome; ICF: idiopathic chronic fatigue.

## Data Availability

The raw data supporting the conclusions of this manuscript will be made available by the authors, without undue reservation, to any qualified researcher.
